# Embryonic Death Is Linked to Maternal Identity in the Leatherback Turtle (*Dermochelys coriacea*)

**DOI:** 10.1371/journal.pone.0021038

**Published:** 2011-06-14

**Authors:** Anthony R. Rafferty, Pilar Santidrián Tomillo, James R. Spotila, Frank V. Paladino, Richard D. Reina

**Affiliations:** 1 School of Biological Sciences, Monash University, Melbourne, Victoria, Australia; 2 Department of Bioscience and Biotechnology, Drexel University, Philadelphia, Pennsylvania, United States of America; 3 Department of Biology, Purdue University, Fort Wayne, Indiana, United States of America; Institute of Marine Research, Norway

## Abstract

Leatherback turtles have an average global hatching success rate of ∼50%, lower than other marine turtle species. Embryonic death has been linked to environmental factors such as precipitation and temperature, although, there is still a lot of variability that remains to be explained. We examined how nesting season, the time of nesting each season, the relative position of each clutch laid by each female each season, maternal identity and associated factors such as reproductive experience of the female (new nester versus remigrant) and period of egg retention between clutches (interclutch interval) affected hatching success and stage of embryonic death in failed eggs of leatherback turtles nesting at Playa Grande, Costa Rica. Data were collected during five nesting seasons from 2004/05 to 2008/09. Mean hatching success was 50.4%. Nesting season significantly influenced hatching success in addition to early and late stage embryonic death. Neither clutch position nor nesting time during the season had a significant affect on hatching success or the stage of embryonic death. Some leatherback females consistently produced nests with higher hatching success rates than others. Remigrant females arrived earlier to nest, produced more clutches and had higher rates of hatching success than new nesters. Reproductive experience did not affect stage of death or the duration of the interclutch interval. The length of interclutch interval had a significant affect on the proportion of eggs that failed in each clutch and the developmental stage they died at. Intrinsic factors such as maternal identity are playing a role in affecting embryonic death in the leatherback turtle.

## Introduction

Leatherback sea turtles (*Dermochelys coriacea*) are large, pelagic reptiles that undertake long oceanic migrations [1], [2], [3]. They were once thought to be the most abundant of the 7 living sea turtle species but have suffered substantial population declines in many parts of the world [4]. The reduction in the global leatherback population has been estimated at ∼67%, with eastern Pacific colonies suffering a 90% decrease in the last two decades [5], [6]. As a result, the population at Parque Nacional Marino Las Baulas (PNMB) in Costa Rica currently faces extinction [6], [7].

Declining leatherback numbers have been attributed to anthropogenic factors such as fishing practices causing unsustainable adult mortality, egg poaching on nesting beaches, and habitat degradation [6], [8], [9]. Natural processes such as beach location, tidal inundation, bacterial and fungal attack and low hatching success are also thought to play a role in their demise [10], [11], [12], [13], [14]. Leatherbacks are now classified as critically endangered by the Species Survival Commission (IUCN)[15] and are the only extant species within the family Dermochelyidae.

Leatherback females return to their natal beaches to nest every 3–4 years once sexually mature [16], [17] to lay an average 7 clutches of eggs during a nesting season. Nesting success and hatchling output varies within and between nesting seasons [18]. Generally during a nesting period, a gravid female will return every 9–10 days to lay an average of 65 eggs per clutch. [17], [19]. They are unusual among oviparous amniotes because they have the highest clutch frequency and produce the biggest mass of eggs during each reproductive cycle using an evolutionary strategy thought to overcome low hatching success [20]. Leatherbacks have an average global hatching success rate of ∼50%, lower than all other marine turtle species, which have hatching success rates in excess of 80% [10], [16]. Low hatching success is an artefact of reduced genetic diversity in declining avian populations [21]. However, this is unlikely the case for leatherback turtles because the worlds largest stable and increasing leatherback rookeries in Western Africa [22], [23] still have relatively low hatching success rates (33–39% at Awala–Yalimapo beach in French Guiana [24]; 10.6–56.0% in Suriname [25] and 67–69% in Gabon [26], [27]).

Infertility causes low hatching success in some avian species [28], [29]. However, this is not the case for leatherback turtles, with low hatching success attributed to high levels of early stage embryonic death [10]. At PNMB embryonic death has not been linked to extrinsic environmental factors that include temperature and oxygen levels within relocated nests [30], [31]. However, in natural nests environmental variables such as precipitation and temperature are believed to play a role (Santidrián Tomillo et al. unpublished data), [18]. The high levels of variation and low coefficients of the relationships between embryonic death and temperature in previous studies suggest that factors other than temperature are involved [18]. Investigations including intrinsic factors such as the effects of maternal identity are only cursory and need further investigation [18], [20], [31]. However, it is known that some leatherback females are better mothers than others [10].

Maternal effects are defined as the underlying pressure exerted by the maternal genotype or phenotype that influences the offspring phenotype [32]. Maternal reproductive investment of turtles is directly represented by the allocation of energy and nutrients because there is no post-hatching parental care [33]. During reproduction, females produce amniotic eggs that consist of yolk and albumin encased in a proteinaceous shell. Approximately 7 days after ovulation, sea turtle embryos enter a state of pre-ovipositional developmental arrest when they become gastrulae inside the oviducts of the female [34], [35]. The mechanisms that cause arrest are currently unknown. Arrest persists for the remaining period of egg retention and ensures that membrane adhesion does not occur inside the egg prior to oviposition, thus preventing movement-induced mortality during laying [36]. Bell et al. (2003) found that the majority of leatherback embryos dying were at Miller's (1985) stage 6 (oviposition) of development. This suggests that embryos died as arrested gastrulae before they were laid or because they failed to break arrest and continue developing afterwards.

Gastrulation is evidently a very important developmental checkpoint for many species laying cleidoic eggs. In some reptilian species, if embryos have to remain as arrested gastrulae during periods of extended egg retention it can result in developmental deformities and even death of the embryos [37]. In addition, pre-ovipositional death of avian embryos occurs if egg development exceeds, or has not reached gastrulation at the time of oviposition [28].

Extended egg retention and hence periods of arrest during interclutch intervals have been recorded at 63 days in the olive ridley turtle (*Lepidochelys olivacea*) [38]. Other species of marine turtle have shorter interclutch intervals and therefore embryos are in arrest for a shorter time. Leatherback interclutch intervals range from 7–14 days [17], with older females having shorter interclutch intervals than younger or first-time nesters (Reina, unpublished data). This raises the question whether the duration of retention and arrest has an affect on embryo survival and if the reproductive experience of the female may also play a role.

The objectives of this study were to investigate whether the maternal identity, the year that each female nests, the position of each clutch relative to other clutches laid that season and the time of nesting each season were affecting hatching success and stage of death of embryos. In addition we wanted to uncover whether there was a difference in hatching success levels and stage of death of embryos between remigrants and new nesters, and between females with different interclutch intervals. Identifying the main cause of early stage embryonic mortality in leatherback nests at PNMB will not just tell us more about the life history patterns of these animals, but also offer an insight into the developmental processes occurring inside the cleidoic egg. This will prove beneficial to those studying reduced hatching success in reptiles and birds. In addition, it may be possible to increase hatching success rates by improving early stage embryonic survival and therefore increase the number of hatchlings entering waning populations.

## Methods

### Ethics statement

This study was conducted under MINAET permits (ACT-OR-052; ACT-OR-051; ACT-PNMB-010-2008; ACT-PNMB-009-2008) and was approved by the Animal Care Committee of Drexel University (IACUC: 18467; 16532).

### Study site and data collection

Data for this study was collected as part of a continuing beach monitoring project at Parque Nacional Marino Las Baulas in the Guanacaste province of Costa Rica (10°20′N, 85°51′W). We used the results of nightly beach surveys conducted during five nesting seasons between October and February from 2004/05 to 2008/09. Approximately 90% of all nesting activity was encountered during these patrols and nesting data was collected for each female using methods previously described [17], [39]. Females were identified as new or remigrant by the absence or presence of PIT tags applied in a previous nesting season. Untagged turtles had a tag applied into the muscle of the shoulder using standard techniques [40]. Oviposition was observed during successful nesting events, eggs were counted and nests were marked with a thermocouple as eggs were laid. The nest location was triangulated to enable subsequent excavation for determination of developmental success.

### Staging dead embryos

Each recorded nest was excavated two days after first hatchling emergence and the contents of all unhatched eggs were examined. The dead embryos were staged using the field-staging method developed by Leslie et al. (1996), which relates to specific developmental stages of Miller's (1985) 31-stage developmental chronology for marine turtles. This criterion corresponds to the presence or absence of an embryo and its associated morphology [10], [30]. Using these guidelines, “field stage 0” was classified as Miller's (1985) stage 12 and below with no embryo or blood vessels visible, with a maximum period of growth of 4 days post oviposition. “Field stage 1” was between Miller's (1985) stages 13–16 and was identified by the presence of an unpigmented embryo <10 mm in length with blood vessels visible, with period of growth from 4–9 days. “Field stage 2” was between Miller's (1985) stages 17–23 and was determined from the presence of an embryo 10–20 mm in length with pigmented eyes, with period of growth from 9–24 days. “Field stage 3” was between Miller's stages 24 and 31 and included an embryo >20 mm long that was fully pigmented, with growth time from 24–60 days. Some previous studies also assigned eggs to an “unknown” category due to decomposition [10]. However for the purpose of this study, any decomposing eggs not exhibiting an embryo or evidence of blood vessel development within the egg was classified as field stage 0 based on the definitions outlined above. Therefore no eggs were assigned to an unknown category for this study.

### Data analysis

Females nesting more than once each season were included in the analysis. Hatching success was calculated as a percentage of the total number of eggs laid in each clutch. Embryonic mortality at each field stage was calculated for each clutch as a percentage of the total amount of eggs that failed in the clutch. Clutch position was identified as its numerical arrangement within successive clutches laid by each specific female in a single season (i.e. clutch position of the first recorded clutch of the season was 1, the second clutch laid by that same female was 2, and so on). Females' reproductive experience was defined as either ‘new’ or ‘remigrant’ from the PIT tag records. The interclutch interval (number of days) between successive clutches for each female was determined using the methods outlined by Reina et al. (2002). Data was included in the analysis if the interclutch interval was between 7–13 days (inclusive). Successful ovulation and full calcification of the eggshell takes a minimum of 6 days [35], so it is not possible for females to lay successive clutches in less than 7 days. In addition, it is possible that females may nest twice within a 14-day period based on this assumption. Therefore, the longest interclutch interval considered was 13 days, with interclutch intervals greater than this likely resulting from an intervening, unobserved nest. The time of nesting during each season was calculated using weekly 7-day intervals beginning when the first nest that season was laid. Female arrival time was defined as the nesting week in the season when each female laid her first nest.

In order to determine whether embryonic mortality occurred equally at all stages the mean log transformed percent mortality values were compared between each field stage using a one-way analysis of variance (ANOVA) and a Tukey's HSD test. Linear mixed effect models (using the ‘LME’ function in the ‘nlme’ package of R statistical package) were used to test the effects of female identity, season, clutch position and time of nesting on various different variables. They were most appropriate because they identify the variation that exists between and among variables such as female identity [20], [41], [42]. Female identity, clutch position and time of nesting were treated as random effects in each model. Hatching success, clutch number and clutch size were treated as response variables, while season, interclutch interval, reproductive status and stage of embryonic death were treated as fixed predictor variables. Models were compared using the Akaike Information Criterion (AIC). P-values and the log-likelihood test statistic (denoted as ‘L’ in text) are reported for these tests. Linear regression was also used in addition to the mixed model to show the relationship between interclutch interval and percent hatching success. The coefficient of determination (R^2^ value) is reported for this test to allow interpretation of variation explained by the mixed model. Finally, ANOVA was used to identify whether female reproductive status affected initial arrival time of females each season and the number of clutches laid.

All data analysis was conducted in R, statistical package 2.11.0 (R Development Core Team 2010) using log transformed percent values. Statistical significance was accepted at the 0.05 level and all values are reported as the mean ± standard error.

## Results

### Stages of embryonic mortality

We analysed 694 clutches laid by 207 leatherback females during the five nesting seasons. In total, 39,690 eggs were laid of which 19,701 failed to hatch. Of the failed eggs, 11,728 (57.2%) failed at stage 0 and 2,100 (10.7%), 694 (3.5%) and 5,629 (28.6%) eggs failed at stages 1, 2 and 3 respectively. Results of the ANOVA and Tukey's test indicated a significant difference between all mean log transformed percent mortality values for each field stage (df = 3, F = 959.24, p<0.01). Mean hatching success was 50.4%.

### Clutch effects

Three females laid an eighth clutch and one female laid a ninth clutch. Due to small samples sizes, these were removed for analysis. Remigrant females laid significantly more clutches than new nesters (2.5±0.1 and 2.0±0.1 clutches respectively; L = 10.98, p<0.01). However, mean clutch size did not differ significantly between remigrant (58.8±0.61 eggs) and new (57.4±0.78 eggs) females (L = 0.22, p = 0.64). In addition, clutch position had no significant affect on hatching success within a clutch (L<0.01, p = 0.99). Although not significant, there was a general trend in the data for the mean hatching success rate to decrease with each successive clutch (Table 1). The mixed model that excluded clutch position as an effect was significantly better at explaining variance in the stage of embryonic death than the model that included clutch position (L = 28.21, p<0.01) so, clutch position also did not significantly affect stage of embryonic death.

**Table 1:** Mean percent hatching success (± standard error) and number of clutches in relation to clutch chronological position.

	Clutch identity						
	Clutch 1	Clutch 2	Clutch 3	Clutch 4	Clutch 5	Clutch 6	Clutch 7
Mean percent hatching success	51.25±1.76	52.78±2.00	48.94±2.46	51.98±3.11	45.44±3.80	43.50±5.53	32.97±8.60
Number of clutches	244	181	115	75	43	23	9

### Female effects

There was a significant relationship between maternal identity and the hatching success in each clutch. (L = 56.03, p<0.01). Reproductive status of the female also significantly affected hatching success (L = 8.82, p<0.01), with a mean hatching success rate for remigrants of 52.40±1.18% (127 females, 516 clutches) and 44.22±2.01% for new nesters (80 females, 178 clutches). The interclutch interval did not differ significantly between remigrant (9.60±0.08 days) and new (9.69±0.09 days) females (L = 1.98, p = 0.16). There was a significant increase in mean percent hatching success with an increase in the duration of the interclutch interval (L = 10.54, p<0.01, R^2^ = 0.04).

**Figure 1:**
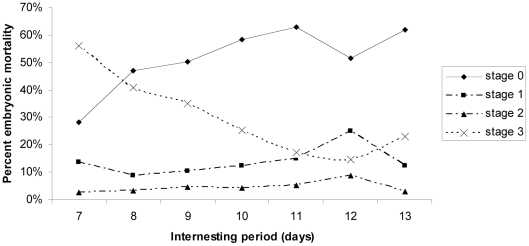
Relationship between the mean percentage of embryos dying at each field stage and interclutch interval (days).

Females' remigrant or new reproductive status did not significantly affect the stage at which embryos died (L = 0.64, p = 0.89), but the interclutch interval did (L = 54.83, p<0.01). Mixed model reconstruction to include stage of death as a subset showed that Stage 0 (L = 4.68, p = 0.03), Stage 1 (L = 8.41, p<0.01) and Stage 3 embryonic death (L = 12.45, p<0.01) were all significantly affected by the interclutch interval, while Stage 2 (L = 3.71, p = 0.05) was not (Figure 1). Embryonic mortality during stage 3 was highest when the interclutch interval was 7 days (56±15.04%) and lowest at 12 days (14.7±4.29%). The reverse occurred in the number of embryos dying during stage 0 with mortality lowest when the interclutch interval was 7 days (28.2±10.91%) and highest at 11 days (62.8±3.48%) before a decline in mortality when the interclutch interval was 12 days (51.67±5.45%). Embryonic mortality increased again at both stages 0 and 3 when the interclutch interval was 13 days (62±6.50% and 23±7.40% respectively) (Figure 1). Average mortality values during stages 1 and 2 appeared to remain relatively constant, although there was a rise in mortality at an interclutch interval of 12 days (24.80±4.09% and 8.80±2.46% respectively) (Figure 1).

### Season effects

**Figure 2:**
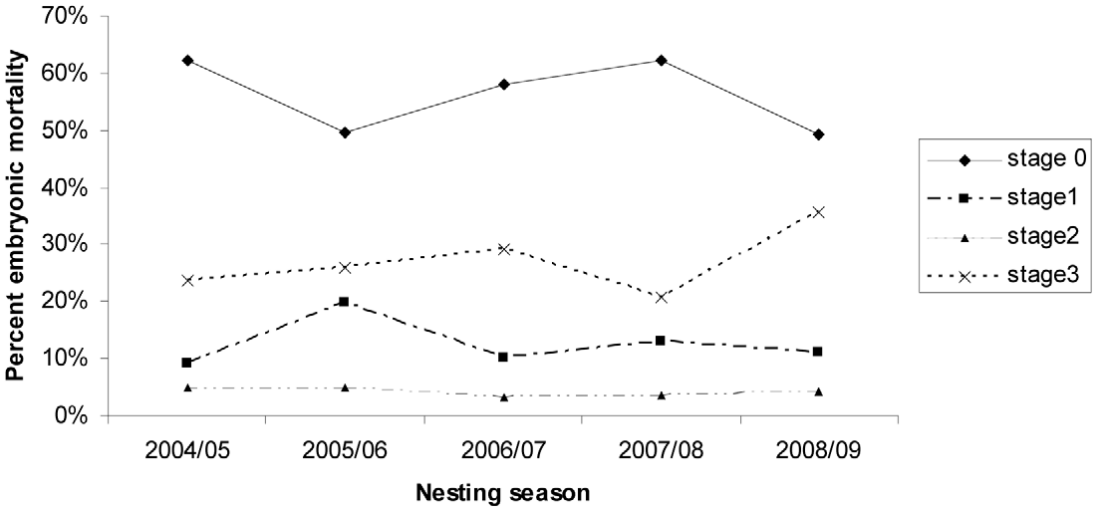
Relationship between the mean percentage of embryos dying at each field stage and nesting season (2004/05–2008/09).

Season significantly affected hatching success (L = 90.98, p<0.01). Lowest hatching success was during the 2006/07 nesting season with a mean percent hatching success of 38.16±2.07%. The highest hatching success was during the 2007/08 season with a mean percentage of 64.61±1.6% (Table 2). The season also significantly affected the stage of embryonic death (L = 105.51, p<0.01) with stages 0, 1 and 3 being significantly affected (L = 26.98, p<0.01; L = 43.84, p<0.01 and L = 16.01, p<0.01 respectively), whereas stage 2 was not (L = 8.00, p = 0.09). Across all seasons, mean percent embryonic mortality was consistently higher at stage 0 (56.2±1.0%) than all other stages (Figure 2). This was then followed by stage 3 (26.4±1.0%), stage 1 (13.1±0.6%) and stage 2 (4.2±0.3%), (Figure 2).

**Table 2:** Mean percent hatching success (± standard error) and number of clutches per season.

	Season				
	2004/05	2005/06	2006/07	2007/08	2008/09
Mean percent hatching success	38.83±2.43	55.65±2.00	38.16±2.07	64.61±1.60	49.64±2.68
Number of clutches	128	171	129	158	108

**Figure 3:**
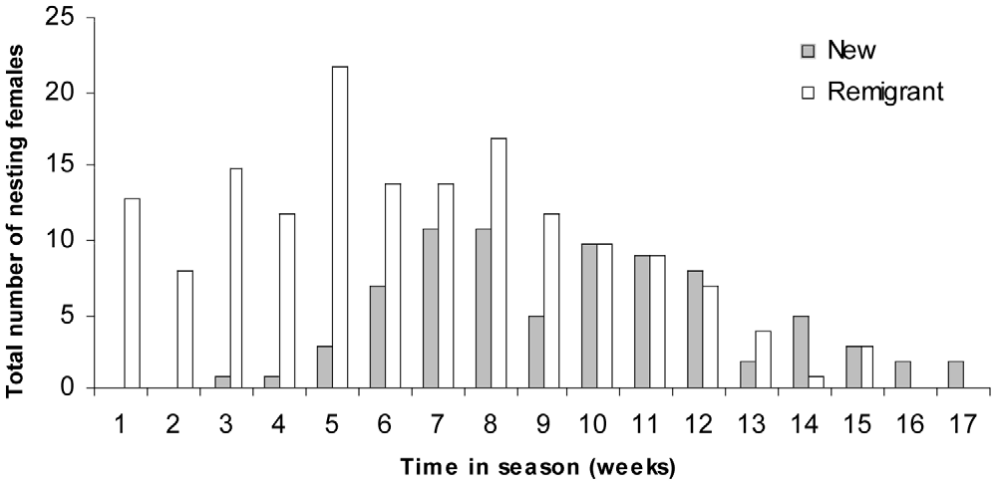
Total number of new and remigrant females nesting each week between 2004/05 and 2008/09.

The nesting week during a season had no significant effect on hatching success within a clutch (L<0.01, p = 9.99). In addition, the mixed model that excluded nesting week as an effect was significantly better at explaining variance in the stage of embryonic death than the model that included it (L = 173.17, p<0.01) so, nesting week did not significantly affect stage of embryonic death. Nesting week also had no significant affect on the duration of the interclutch interval (L<0.01, p<0.01). However the interclutch interval was significantly affected by the status of the female, with remigrant females arriving earlier in the season to nest than new nesters (df = 1, F = 47.28, p<0.01; figure 3). Across all 5 nesting seasons, mean arrival time was 6.5±0.3 weeks for remigrant females and 9.7±0.4 weeks for new nesters (figure 3).

## Discussion

During the study period, remigrant females arrived earlier in the season to nest, produced more clutches and had an overall higher rate of hatching success than new nesters. Maternal identity, maternal reproductive experience, interclutch interval and nesting season significantly affected hatching success of clutches, whereas clutch position and time of nesting did not. This agrees with previous research that showed hatchling production varies between nesting seasons at PNMB [18]. However, it differs from the findings presented by Garrett et al. (2010) showing that inter-clutch variation exists in hatching success among females in St Croix, and that a decrease in hatching success occurs with nesting season progression [18]. This latter result may have been an artefact of including 3 wet seasons and 2 dry seasons in this analysis, reducing the impacts that dry El Nino years have on hatching success, which generally result in increased mortality toward the end of the season (Santidrián Tomillo et al. unpublished data). This theory is supported by the apparent trend in the data, which, although it is not significant, suggests that hatching success decreases as the season progresses and each successive clutch is laid. Using an equal number of wet and dry seasons in future studies may accurately reflect this and is something that needs to be taken into consideration when conducting further investigations.

There was also a significant relationship between nesting season and interclutch interval, and the stages when embryos died. Clutch position and time of nesting had no significant affect on the proportion of embryos dying at different stages in this study. This suggests that within a given season embryos of certain females tend to die at a specific stage of development. The concept of ‘good’ and ‘poor’ mothers suggested by the data of Bell et al. (2003) seems to be explained at least in part by this maternal effect on the distribution of embryonic death in a clutch.

Some leatherback females consistently produce nests with higher hatching success rates than others [10]. The majority of embryonic death between the 2004/05 and 2008/09 nesting seasons occurred at field stage 0. This is also consistent with Bell et al. (2003) that most embryonic death in leatherback nests occurs primarily during early stages of development prior to and at Miller's (1985) stage 6. This pattern of embryonic mortality is contrary to that observed in St. Croix where most embryonic death occurs during late stages of development [16]. Regional variation in rates of hatching success of leatherbacks occurs. Investigations on leatherback nesting beaches include Tongaland, South Africa, 76% [43], Suriname, 50% [44], Rantau Abang, Malaysia, <56.4% [45], and Tortuguero, Costa Rica, 53.2% [13]. Variations in the distances travelled by females between breeding and feeding locations also exist in both the Atlantic and Pacific Oceans [1], [2], [3]. Distances and duration of nesting migrations can affect the health of mothers, thus causing variations in the amount of resources available for egg allocation and individual fecundity [46].

In turtles, maternal reproductive investment is directly represented by the allocation of energy and nutrients because there is no post-hatching parental care [33]. During reproduction, females produce amniotic eggs that consist of yolk and albumin encased in a proteinaceous shell. The proteins produced in the mother's oviduct largely dictate the internal incubation environment of the egg [47], [48]. Reptiles are known to produce low molecular weight proteins in the oviduct that affect embryonic development [49]. In addition to these maternal influences on the embryo, the embryo is also capable of producing factors that affect oviducal activities [49]. Growth factors secreted by American alligators (*Alligator mississippiensis*) are present in the oviducal secretions of gravid females and become incorporated into egg albumin and affect embryonic development [47], [49], [50]. Preliminary work on turtles has identified similar growth factors in slider turtle (*Trachemys scripta*) plasma [51]. Maternal effects have been used to describe differences in the growth rate and righting response of snapping turtle (*Chelydra serpentine*) hatchlings [39], [52]. It is therefore plausible that female turtles directly influence the development of their embryos inside the oviduct and possibly after oviposition.

In addition to water and solids, mothers supply the albumin of eggs with factors necessary for development [20], [53]. Insulin-like growth factors I and II have been identified as products that are secreted by the reptilian reproductive tract [47], [51]. These factors influence development in a multitude of different species [54], [55]. Binding proteins associated with IGFs (IGFBP) are genetically induced by hypoxia [56], [57], [58]. Embryonic metabolism is believed to arrest in response to hypoxia in turtle oviducts [37], [59], [60], [61], [62], [63], perhaps through the activity of IGFBP. Although this remains to be investigated, it may be plausible that the activity of growth factors present in the egg albumin is affected by environmental variables such as oxygen both inside the female and the nest. Embryos of some females may be more developmentally sensitive to environmental conditions than others [30]. Environmental variability driven by the El Niño Southern Oscillation affects hatching and emergence success of leatherback turtles in Costa Rica (Santidrián Tomillo et al. unpublished data). Evaluating maternal environmental effects in an ecological context has demonstrated that mothers may provide phenotypic adaptation to local environmental conditions [64], [65], [66].

There is no significant difference between the interclutch intervals of remigrant and new females. This is consistent with the result that the interclutch interval does not change as the season progresses, regardless of the different peak nesting periods for both new and remigrant females. New females have lower rates of hatching success than remigrant females, but female status does not affect the stage at which embryos die. This suggests that it is the duration of exposure to the maternal oviducal environment that is affecting the stage of embryonic death rather than the experience of the female. Embryonic death at field stage 3 steadily declines as the interclutch interval gets longer and approaches 12 days. Although it remains to be tested, perhaps additional time spent in the hypoxic environment of the oviduct conditions the embryos in some way to later withstand hypoxia in the nest. The maternal provisioning of glycogen metabolites to her embryos can affect their ability to survive low oxygen conditions in *C. elegans* [66]. Perhaps increased allocation of glycogen, or other oviducal factors, by some females *in utero* prevents embryonic mortality when oxygen tensions decrease in the nest during the final stages of incubation. This may explain why late stage embryonic mortality and hatching success is not significantly affected by hypoxia at PMNB [30], [31]. Changes in oxygen and carbon dioxide partial pressures occur during the incubation period in response to the collective development of embryos [30], [31], [67]. Hypoxia and hypercapnia occur during the final stages of incubation between days 50 and 60 with declines in oxygen tensions and increases in carbon dioxide levels occurring from day 35 at PNMB [30]. The second half of development is the period when embryonic growth and development is most sensitive to changes in respiratory gas concentrations [68].

Olive ridley turtles (*Lepidochelys olivacea*) naturally have extended interclutch intervals of up to 63 days during which, embryos are maintained in a state of arrested development and subsequently resume development after oviposition [38]. This is likely a reproductive strategy this species employs to enable mass arribada nesting [69]. Delayed nesting and extended periods of egg retention have also been reported in various freshwater turtle species including *Deirochelys reticularia* (chicken turtle), *Kinosternon subrunrum* (eastern mud turtle) and *Trachemys scripta* (red-eared slider) [70], [71]. It would be interesting for future research to identify how embryos are maintained in this arrested state for such long periods and how it might affect subsequent embryonic development.

In conclusion, in addition to seasonal effects on hatching success in leatherback turtles, some females are better mothers than others. The study of how intrinsic factors such as maternal effects impact upon sea turtle embryonic death is a new field and at this stage we can only speculate mechanisms that may be actively affecting embryonic mortality. We hypothesize that mothers may be affecting the development of their young by producing factors that become incorporated in the internal environment of the egg and infer different levels of developmental sensitivity to environmental conditions. By identifying the causes of embryonic death in the leatherback turtle, we can use this information as a baseline understanding of embryonic mortality in not just other chelonian species and reptiles in general, but also other egg laying animals. It may also allow strategies to be put into place that increase hatching success and production in this critically endangered species.
